# Safflower Polysaccharide Inhibits AOM/DSS-Induced Mice Colorectal Cancer Through the Regulation of Macrophage Polarization

**DOI:** 10.3389/fphar.2021.761641

**Published:** 2021-10-22

**Authors:** Qun Wang, Yun Huang, Min Jia, Dong Lu, Hong-Wei Zhang, Dan Huang, San-Hong Liu, Chao Lv

**Affiliations:** ^1^ Institute of Interdisciplinary Integrative Medicine Research, Shanghai University of Traditional Chinese Medicine, Shanghai, China; ^2^ College of Pharmaceutical Sciences, Zhejiang Chinese Medical University, Hangzhou, China; ^3^ Department of Pathology, Fudan University Shanghai Cancer Center, Shanghai, China

**Keywords:** safflower polysaccharide, colorectal cancer, apoptosis, macrophages, NF-κB

## Abstract

Safflower polysaccharide (SPS) is one of the active fractions extracted from safflower petals (*Carthamus tinctorius L*.) which has been reported to possess antitumor and immune control roles. However, its antitumor mechanisms by regulating the immune pathway remain barely understood. In this study, a mouse model was established by azoxymethane (AOM)/dextran sodium sulfate (DSS) to evaluate the antitumor effect of SPS on colorectal cancer (CRC). The results showed that 50 mg/kg SPS-1, an active fraction isolated from SPS, could significantly inhibit CRC induced by AOM/DSS and changed the polarization of macrophages to the M1 phenotype. Meanwhile, SPS-1 treatment significantly alleviated the characteristic AOM/DSS-induced pathological symptoms, in terms of decreasing the nucleoplasmic ratio, nuclear polarity extinction, and gland hyperplasia. However, the results *in vitro* showed that SPS-1 did not directly inhibit the growth of CRC cells but could upregulate the NF-κB signal and trigger M1 macrophage transformation. Thus, the condition medium (CM) of Mφ pretreated with SPS-1 was used against CRC cells. As expected, SPS-1–activated Raw 264.7 markedly exhibited antitumor effects by inhibiting cell proliferation and suppressing cell colony formation. In addition, SPS-1–activated Raw 264.7 could also induce CRC cell apoptosis by upregulating the levels of tumor necrosis factor-α (TNF-α) and nitric oxide (NO). Further results suggested that SPS-1–induced transition of the macrophage phenotype could be suppressed by an NF-κB inhibitor, PDTC. Moreover, SPS-1–activated Raw 264.7 inhibiting CRC cell proliferation and inducing apoptosis were also rescued by PDTC. Taken together, all results suggested that SPS-1 could be a therapeutic option for the prevention and treatment of CRC.

## Introduction

Colorectal cancer (CRC) is the third most common malignant tumor in the world, with morbidity and mortality second to gastric and liver cancer, respectively (Li et al., 2013; Mármol et al., 2017). In 2020, there were 1.93 million new cases of colon cancer, and 935,173 people died of colorectal cancer–related diseases (Siegel.et al., 2020). According to previous reports, most CRC cases are closely associated with environmental factors including chemicals and dietary causing genetic instabilities in intestinal cells, specific intestinal symbiont, and pathogen, and only about 20% of CRC cases exhibit familial hereditary properties (Jasperson et al., 2010; Valle, 2017; Jackson and Theiss, 2020). Epidemiological data also show that chronic intestinal inflammation is the most important underlying etiologies of carcinogenesis. The mechanism may be to cause defects in the host’s antitumor immune system and promote the cancerous process (Jackson and Theiss, 2020).

The early signs of CRC are not obvious until abdominal pain and blood in the stool can be detected. Patients with advanced conditions like anemia and weight loss can only be treated with surgery (Ladabaum et al., 2020). In addition, the tolerance and toxicity of chemotherapeutics limit their clinical application (Kaufmann et al., 2019). Therefore, to prevent colitis-related CRC, the discovery and development of drugs with small side effects and low tolerability are the current priorities. When evaluating the effects of drug candidates on CRC, it is crucial to select a suitable tumor model of CRC. According to short formation time, tumor stability, and similar characteristics to human CRC, the AOM/DSS model is the most suitable for studying the occurrence and development of CRC (Tanaka et al., 2003; Venkatachalam et al., 2020).

Natural polysaccharides possess multiple therapeutic characteristics, including antitumor, anti-inflammation, and immunomodulation (Zhao et al., 2020). Currently, polysaccharide drugs including chondroitin sulfate, ginseng polysaccharide, and lentinan polysaccharide have been widely applied in cancer therapy (Wang et al., 2016; Zheng et al., 2020). Safflower polysaccharide (SPS), one of the active components of safflower, has antitumor and immune regulation effects (Ando et al., 2002). According to previous studies, SPS could significantly inhibit the invasion and migration of various tumor cells, such as gastric cancer and ovarian cancer (Cui et al., 2018). In this study, a polysaccharide fraction (SPS-1) was isolated from SPS; it exhibited no cytotoxicity to colon cancer cells within a dose range of 1–1,000 μg/ml but could induce macrophages to produce TNF-α and NO associated with M1-type macrophages. M1-type tumor-associated macrophages (TAM) can inhibit tumor growth in the microenvironment, while M2-type TAM can facilitate tumor cell survival, proliferation, invasion, angiogenesis, and immunosuppression (Li et al., 2021). Therefore, we explored the antitumor effect and potential mechanisms of SPS-1 on colorectal cancer cells by regulating the polarization of tumor-associated macrophages.

## Materials and Methods

### Materials

Dextran sulfate sodium (DSS) and azoxymethane (AOM) were obtained from Yeasen Biotechnology Co., Ltd. (Shanghai, China). Antibodies of p-IκBα, p-p65, IκBα, p65, and GAPDH were purchased from Cell Signaling Technology (Danvers, MA, United States). Human TNF-α and the IL-6 ELISA kit were obtained from eBioscience (Vienna, Austria). Anti–CD16-PE, CD11c-FITC, CD11b-FITC, F4/80-PE, CD-3-APC, CD80-PE, and CD206-PE were obtained from eBioscience (Minneapolis, MN, United States). The Annexin V-FITC/PI apoptosis detection kit was obtained from BD Biosciences (San Jose, CA, United States). Calcein-AM/PI was acquired from Beyotime (Haimen, China). PDTC was purchased from MedchemExpress (Monmouth Junction, NJ, United States). Safflower polysaccharide (SPS) was obtained from Shanghai Yuanye Bio-Technology Co., Ltd. (Shanghai, China).

### Cell Culture

The cell lines of HCT116, RKO, SW480, LoVo, MC38, and RAW 264.7 were obtained from Cell Bank of Shanghai Institute of Cell Biology, Chinese Academy of Sciences (SIBS, CAS). The RAW 264.7, LoVo, and MC38 cell lines were cultured in DMEM (Hyclone, Logan, UT, United States) with 10% FBS (Biological Industries, Cromwell, CT, United States). The HCT116 cell line was cultured in McCoy’s 5A medium (Gibco, Carlsbad, CA, United States) supplemented with 10% FBS. The SW480 cell line was maintained in the L-15 medium (Gibco, Carlsbad, CA, United States) supplemented with 10% FBS. The RKO cell line was cultured in the MEM medium (Hyclone, Logan, UT, United States) with 10% FBS.

### Detection of Cell Viability

The cell lines of HCT116, RKO, SW480, LoVo, MC38, and RAW 264.7 were planted into 96-well plates (1 × 10^4^ cells/ml) containing different concentrations of SPS-1 or CM. After treatment for 48 h, the effect of SPS-1 on cell viability was measured by Cell Counting Kit-8 (CCK-8, Dojindo), according to the instructions.

### Conditioned Medium Collection

The RAW 264.7 cells were treated with different concentrations of SPS-1 (100, 500, and 1,000 μg/ml, respectively) for 24 h, and then the supernatant was collected and filtered through a 0.22-μm filter. In addition, cells treated by LPS (50 ng/ml) and untreated RAW 264.7 cells were used as a positive control and a negative control, respectively.

### Colony Formation Assay

For colony formation experiment, 1,000 cells (RKO and HCT116) were planted into a 12-well plate and cultured for 7 days in CM. Then cells were stained with Giemsa for colony counting.

### Nitrite Determination, IL-6, and TNF-α Assays

The production of nitric oxide (NO) was detected by using the Griess reaction. The RAW 264.7 cells were planted into 12-well plates (5 × 10^3^ cells/ml) and treated with different concentrations of SPS-1 (100, 500, and 1,000 μg/ml, respectively) for 24 h. Then 50 μl of the culture supernatant was mixed with an equal volume of Griess reagent for 30 min at room temperature. The absorbance was measured by a microplate reader at 450 nm. The levels of IL-6 and TNF-α in RAW 264.7 cells in cell-free supernatant were detected by an ELISA assay kit, according to the instructions (Ebioscience).

### Flow Cytometric Analysis

RAW 264.7 cells were treated with SPS-1 (100, 500, and 1,000 μg/ml, respectively) for 24 h, incubated with PE-labeled CD80, CD16, and CD206 in the dark for 15 min, washed twice with cold PBS, and resuspended with 300 μl PBS. In addition, RKO and HCT116 cells were planted into six-well plates (5 × 10^5^ cells/ml) containing different concentrations of CM. The ratio of the conditioned medium to the normal medium is 3:1, and the medium is changed every 24 h. After 72 h of CM treatment, cells were collected and washed twice with PBS, and then cells were resuspended with 300 μl PBS in tubes. Propidium iodide (PI) or Annexin V was added to the tubes for 20 min according to the corresponding experiment. The result was detected by flow cytometry (BD Biosciences); each analysis was performed using 10,000 cells.

### RNA Isolation and Quantitative Real-Time PCR Analysis

RAW 264.7 cells (2 × 10^6^ cells/six-well plate) were treated with SPS-1 (100, 500, and 1,000 μg/ml, respectively) for 12 h. Total RNA was isolated by RNAiso Plus (Takara, Dalian, China). RNA (1 μg) was reverse-transcribed using the PrimeScript RT Reagent Kit (Takara, Dalian, China). Quantitative RT-PCR was performed using a Roche LightCycler480, and the sequences of the primers are indicated in Table 1. Results were finally normalized to the level of GAPDH gene expression.

**TABLE 1:** Primer sequences for qPCR analysis.

Gene name	Primer sequences
GAPDH	F: 5′-AAC​TTT​GGC​ATT​GTG​GAA​GG-3′
	R: 5′-GGA​TGC​AGG​GAT​GTT​CT-3′
IL-4	F: 5′-GAA​TGT​ACC​AGG​AGC​CAT​ATC-3′
	R: 5′-CTC​AGT​ACT​ACG​AGT​AAT​CCA-3′
IL-23	F: 5′-CAG​CAG​CTC​TCT​CGG​AAT​CT-3′
	R: 5′-TGG​ATA​CGG​GGC​ACA​TTA​TT-3′
TNF-α	F: 5′-CGG​TGC​CTA​TGT​CTC​AGC​CT-3′
	R: 5′-GAG​GGT​CTG​GGC​CAT​AGA​AC-3′
IL-6	F: 5′-TCG​TGG​AAA​TGA​GAA​AAG​AGT​TG-3′
	R: 5′-AGT​GCA​TCA​TCG​TTG​TTC​ATA​CA-3′

### Animal Experiments

All mice were maintained in a pathogen-free facility, and all animal experiments were approved by the Committee on the Ethics of Animal Experiments of the Shanghai University of Traditional Chinese Medicine (SHUTCM). 6-week-old C57 mice were used in animal experiments. The mice were randomly and equally divided into four groups (*n* = 6): control, SPS-1, AOM/DSS, and AOM/DSS+SPS-1. The AOM/DSS and AOM/DSS+SPS-1 groups were injected with 10 mg/kg of AOM intraperitoneally. Seven days after the AOM injection, the mice were given DSS in their drinking water for the third (2% DSS), sixth (1.5% DSS), and ninth (1.5% DSS) week. The same procedure was performed with intraperitoneal normal saline and drinking distilled water instead of the AOM/DSS treatment in the control and SPS-1 groups. The SPS-1 and AOM/DSS+SPS-1 groups were administered by a dosage of 50 mg/kg SPS-1 every day. Mice in AOM/DSS and control groups were treated by gavage equal volume of saline. All mice were sacrificed by cervical dislocation 102 days after the administration of SPS-1.

### Western Blot

RAW 264.7 cells (2 × 10^6^ cells/six-well plate) were treated with SPS (100, 500, and 1,000 μg/ml, respectively) for 30 min to activate the NF-κB signaling pathway. Cells were washed with PBS, and total cells were collected in 100 μl of a lysis buffer. The lysates were centrifuged for 15 min at 4°C, and the supernatant was collected. Proteins were subjected to 10% sodium dodecyl sulfate–polyacrylamide gel electrophoresis (SDS-PAGE) and then transferred to PVDF membranes. The membranes were incubated with primary antibodies at manufacturer’s recommended dilutions at 4°C overnight, and secondary antibodies were added at the recommended dilutions and the incubation continued for another 1 h at room temperature. The protein bands were analyzed with an imaging system (Bio-Rad, United States).

### Statistical Analysis

Statistical analysis was performed with an unpaired t-test when comparing two different groups or one-way ANOVA with Tukey’s multiple comparison tests. The data are expressed as mean ± SEM. *p <* 0.05 was considered significant.

## Results

### Safflower Polysaccharide-1 Suppressed Colorectal Cancer Formation and Growth in Azoxymethane/Dextran Sodium Sulfate-Induced Mice

**FIGURE 1:**
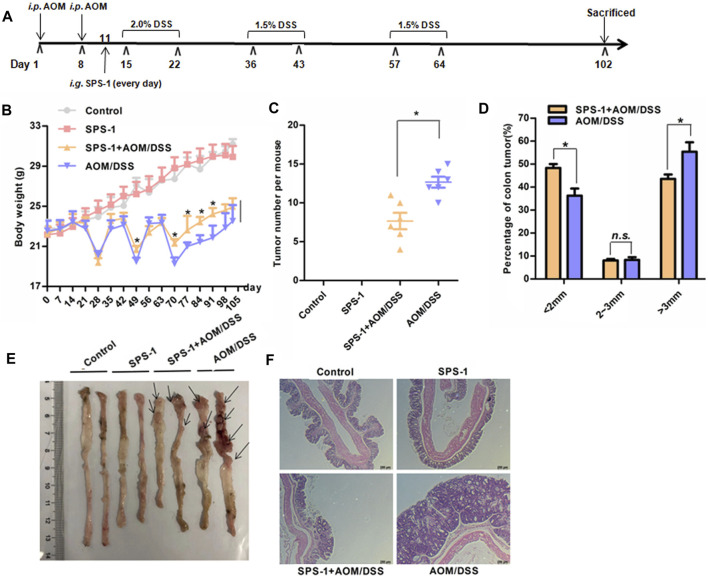
Effects of SPS-1 on AOM/DSS-induced CRC in mice. **(A)** Schematic explanation of the experimental procedure for the AOM/DSS model. The mice body weight **(B)**, the total number **(C)**, and the size distributions of tumors **(D,E)** after 102 days induction. **(F)** Representative gross morphology of mice colon by H&E staining of colon tissues. Data are shown as mean ± SEM. * indicates *p* < 0.05 compared with the AOM/DSS group.

SPS-1 is the major component of SPS, and its chemical properties such as the separation spectrum, monosaccharide composition, average molecular weight, and the infrared spectrum are provided in the supplementary materials (Supplementary Figure S1–S4). To study the antitumor activity of SPS-1, a mouse model was established by AOM/DSS as described previously (Angelou et al., 2018). As shown in Figure 1A, the mice were treated with SPS-1 at a dosage of 50 mg/kg/day *via* gavage. In the model group, we found that mice that freely drank DSS solution for seven consecutive days in the first, second and third cycles showed obvious body weight loss as compared to the control group. In contrast, a significant improvement in weight loss was observed in the SPS-1–treated AOM/DSS group, especially during the third cycle (Figure 1B). Next, we determined the occurrence of CRC in these mice by calculating the number and size distribution of tumors. As shown in Figures 1C,E, the number of tumors in the model group was more than those in the SPS-1–treated AOM/DSS group. Furthermore, the tumor diameters in the model group were mainly distributed > 3 mm (55.39 ± 3.29%), while the diameters in the SPS-1–treated AOM/DSS group were predominantly < 2 mm (55.31 ± 6.39%) (Figures 1D,E). There was no difference between the SPS-1–treated AOM/DSS group and the model group with a diameter of 2–3 mm (Figures 1D,E). Moreover, histologic examination showed that SPS-1 treatment significantly alleviated the characteristic AOM/DSS-induced pathological symptoms, in terms of an increased nucleoplasmic ratio, nuclear polarity extinction, and gland hyperplasia (Figure 1F). These results showed that SPS-1 could significantly reduce the AOM/DSS-induced tumor formation and growth in mice.

### Safflower Polysaccharide-1 Inhibited the Occurrence of Colorectal Cancer Neither by Reducing Intestinal Inflammation nor by Killing Tumor Cells Directly

**FIGURE 2:**
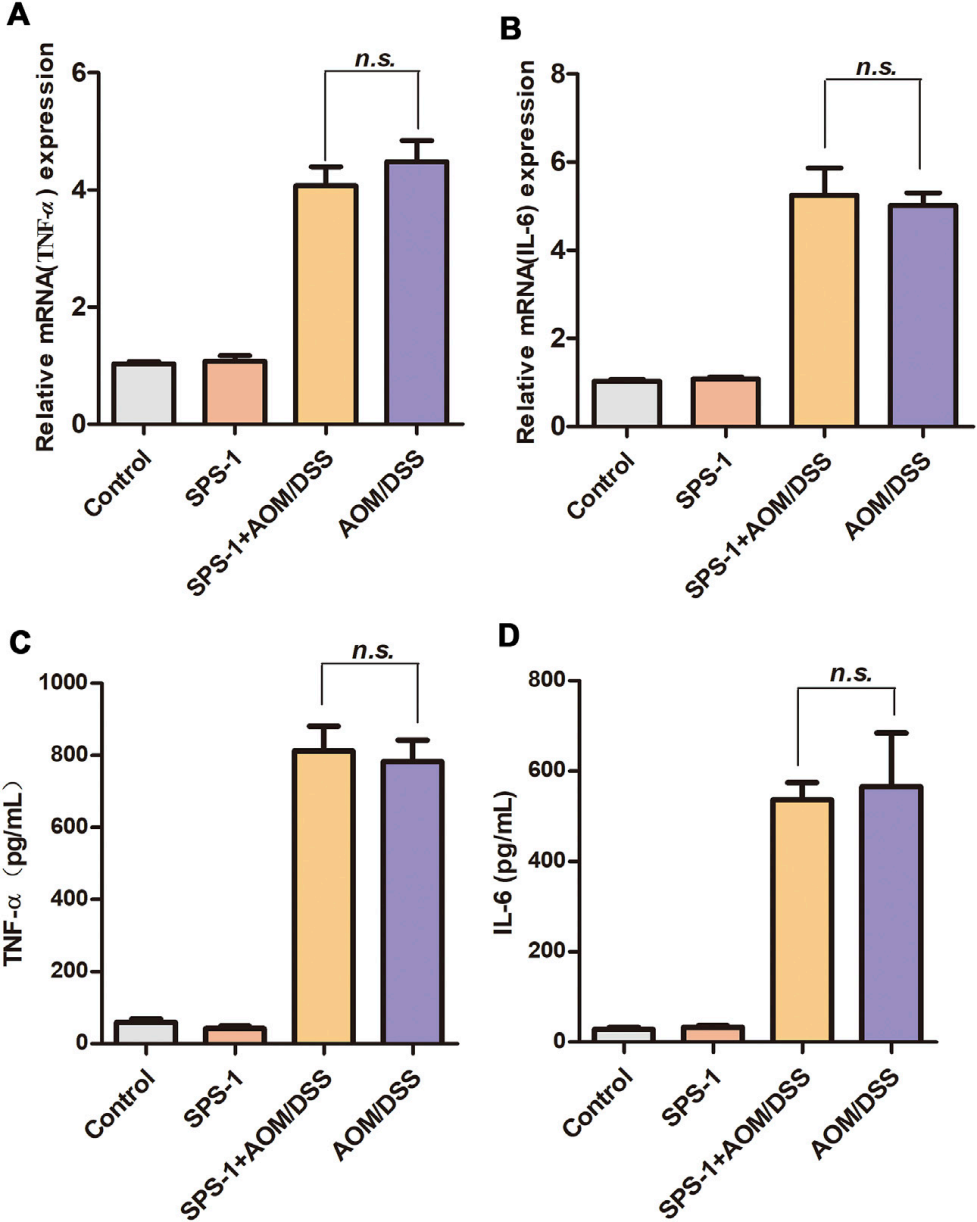
Effect of SPS-1 on AOM/DSS-induced expression of cytokines in colon tissue and serum. **(A,B)** The colon tissue was collected after the experiment, and the mRNA levels of TNF-α and IL-6 were assessed by qRT-PCR. **(C,D)** Blood samples were taken on day 102, and serum levels of TNF-α and IL-6 were assayed by ELISA. Data are shown as mean ± SEM. *n.s.* indicates no significant difference compared with the AOM/DSS group.

To investigate the underlying mechanisms of SPS-1 antitumor effects, qRT-PCR was used to detect the mRNA level of inflammatory cytokines in colon tissue. Compared with the control group, AOM/DSS treatment resulted in a significant increase in the mRNA levels of IL-6 and TNF-α. However, these inflammatory cytokines of the SPS-1–treated AOM/DSS group were not reduced as compared to the model group (Figures 2A,B). Consistent with the qRT-PCR results, there was also no difference in serum levels of IL-6 and TNF-α between the SPS-1–treated AOM/DSS and model groups (Figures 2C,D).

To further explore the underlying antitumor mechanisms of SPS-1, five types of CRC cells were selected to evaluate SPS-1 effects on proliferation. As shown in Table 2, there was no obvious cytotoxicity of SPS-1 (0–1,000 μg/ml) on HCT116, SW480, LoVo, RKO, and MC38 cells. These results showed that SPS-1 neither reduced intestinal inflammation nor directly killed tumor cells to inhibit the occurrence of CRC.

**TABLE 2:** Effects of SPS-1 on cell viability of colorectal cancer cells after 48 h treatment.

SPS-1 (μg/ml)	HCT116 (%)	SW480 (%)	LoVo (%)	RKO (%)	MC38 (%)
0	99.34 ± 1.54	109.12 ± 0.23	101.11 ± 0.34	88.29 ± 1.98	109.07 ± 1.09
100	100.23 ± 0.53	99.21 ± 1.19	99.34 ± 0.49	100.21 ± 2.01	100.12 ± 1.11
200	101.32 ± 2.20	89.99 ± 4.12	98.18 ± 0.02	99.13 ± 0.97	100.39 ± 0.44
400	109.12 ± 0.67	98.39 ± 0.91	99.89 ± 1.20	99.12 ± 2.99	99.54 ± 1.08
800	98.90 ± 1.98	98.86 ± 0.26	99.29 ± 1.22	98.14 ± 0.98	99.10 ± 0.98
1,000	99.87 ± 1.11	101.01 ± 0.30	100.80 ± 0.65	109.99 ± 1.11	100.32 ± 2.11

Each data point represents the mean ± SEM of independent three experiments.

### Safflower Polysaccharide-1 Shifted Macrophage Polarization Toward the M1 Phenotype *In Vivo* and *In Vitro*

**FIGURE 3:**
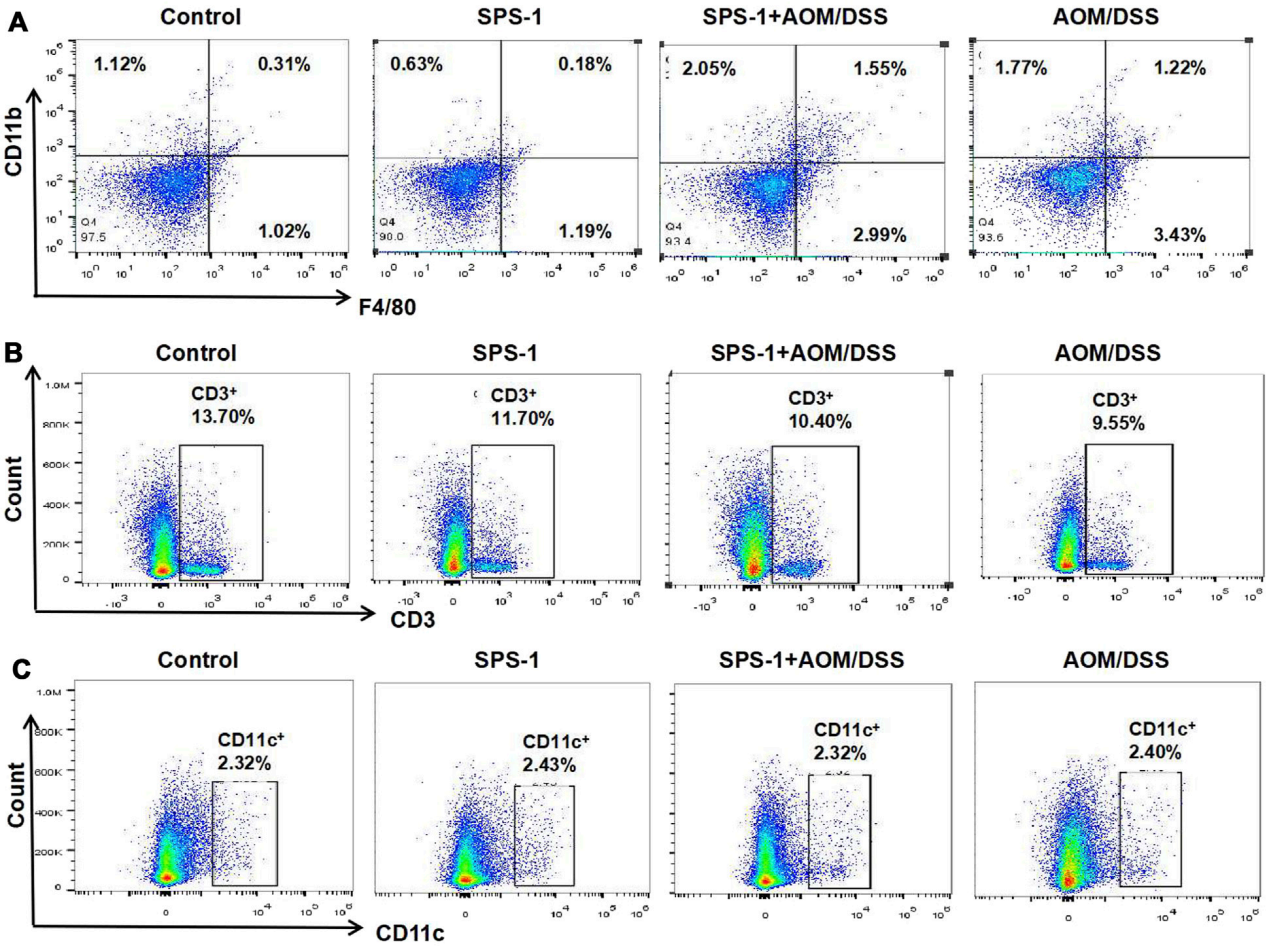
Effect of SPS-1 on the number of **(A)** macrophage, **(B)** T Cells, and **(C)** dendritic cells (DC) in colon tissue of CRC mice. The cells obtained from colon tissue were incubated with different flow cytometric antibodies (CD11b, F4/80, CD3, and CD11c) and detected by flow cytometry.

Based on the above results, we speculated that SPS-1 inhibited CRC by activating the immune system *in vivo*. In this study, the proportion of T cells, dendritic cells (DC), and macrophages in the CRC tissue was determined by flow cytometry. As shown in Figure 3A, macrophages (F480/CD11b) in the colon tissue of the model group were significantly higher than those in the control group. After SPS-1 treatment, the number of macrophages was further increased in the proportion from 1.22% to 1.55%. However, the T-cell levels in colon tissues of the model group and the SPS-1–treated AOM/DSS group were lower than those of the control group (Figure 3B). Also, the proportion of DC cells was not changed in the control group, the SPS-1 group, the AOM/DSS group, and the SPS-1–treated AOM/DSS group (Figure 3C). These results suggested that SPS-1 could selectively activate macrophages *in vivo*.

**FIGURE 4:**
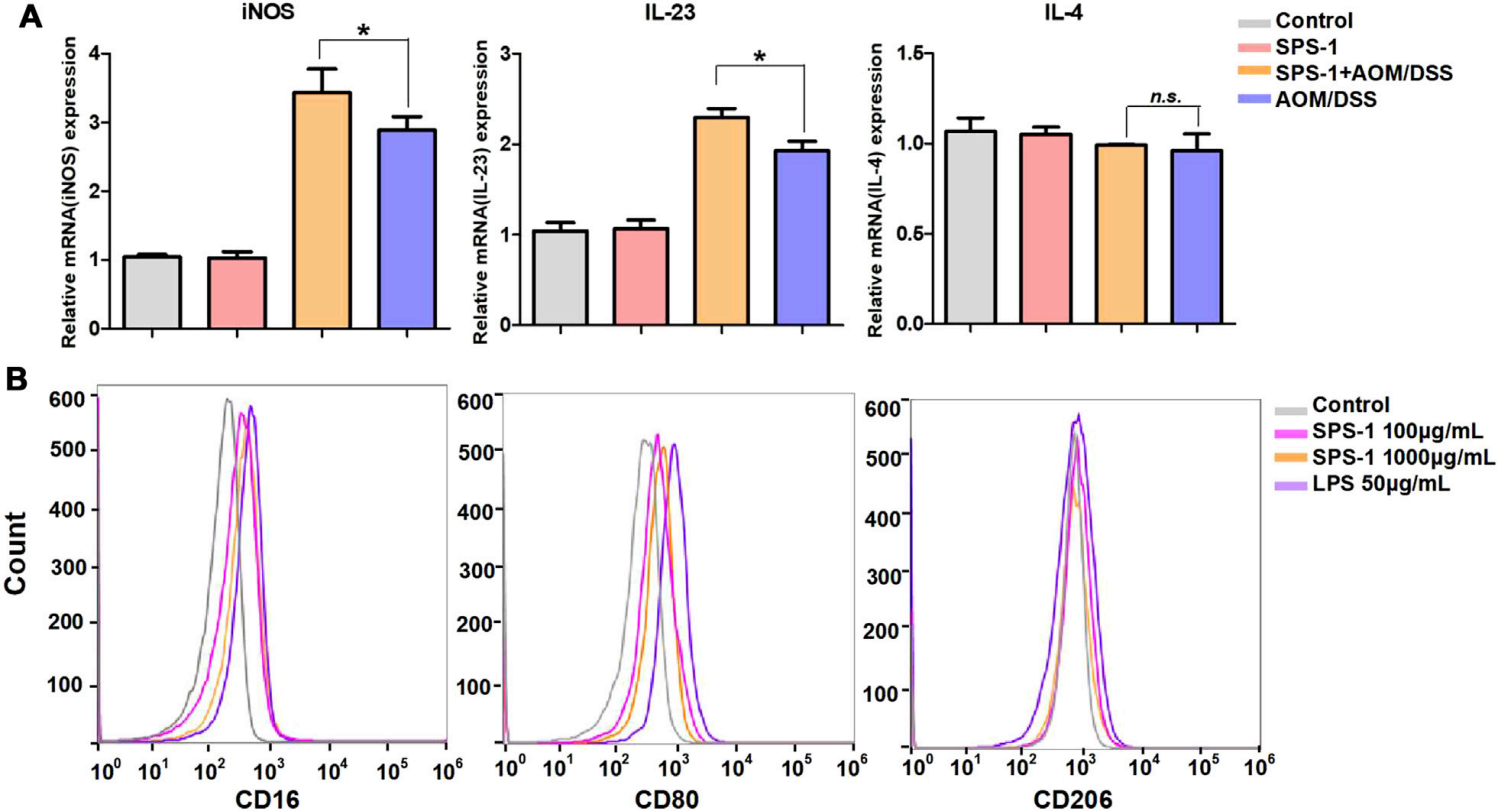
Effect of SPS-1 on macrophage polarization *in vivo* and *in vitro*. **(A)** mRNA levels analysis of M1 markers iNOS, interleukin (IL)-23, and M2 markers IL-4 in colons tissue of the mice. **(B)** CD16, CD80, and CD206 expression on Raw 264.7 stimulated with SPS-1 was analyzed by flow cytometry. Data are shown as mean ± SEM. * indicate *p* < 0.05 compared with the AOM/DSS group.

To describe the differential phenotype of macrophages, CRC tissues were isolated from mice and the expression of M1 and M2 markers was analyzed by real-time PCR. The results showed that the mRNA levels of M1 markers (iNOS and IL-23) were significantly upregulated after SPS-1 treatment, while the M2 marker (IL-4) was not changed (Figure 4A). To further study the relationship between SPS-1 treatment and macrophage polarization, LPS was used to polarize Raw 264.7 cells into M1 type *in vitro*. As shown in Figure 4B, M1 marker genes (CD16 and CD80) were increased and M2 marker genes (CD206) remained unchanged after being treated by SPS-1. These results suggested that SPS-1 treatment could increase the propensity of Mφ polarizing to the M1 phenotype *in vivo* and *in vitro*.

### Safflower Polysaccharide-1 Activated Macrophages to Release TNF-α, IL-6, and Nitric Oxide Through the NF-κB Signaling Pathway

**FIGURE 5:**
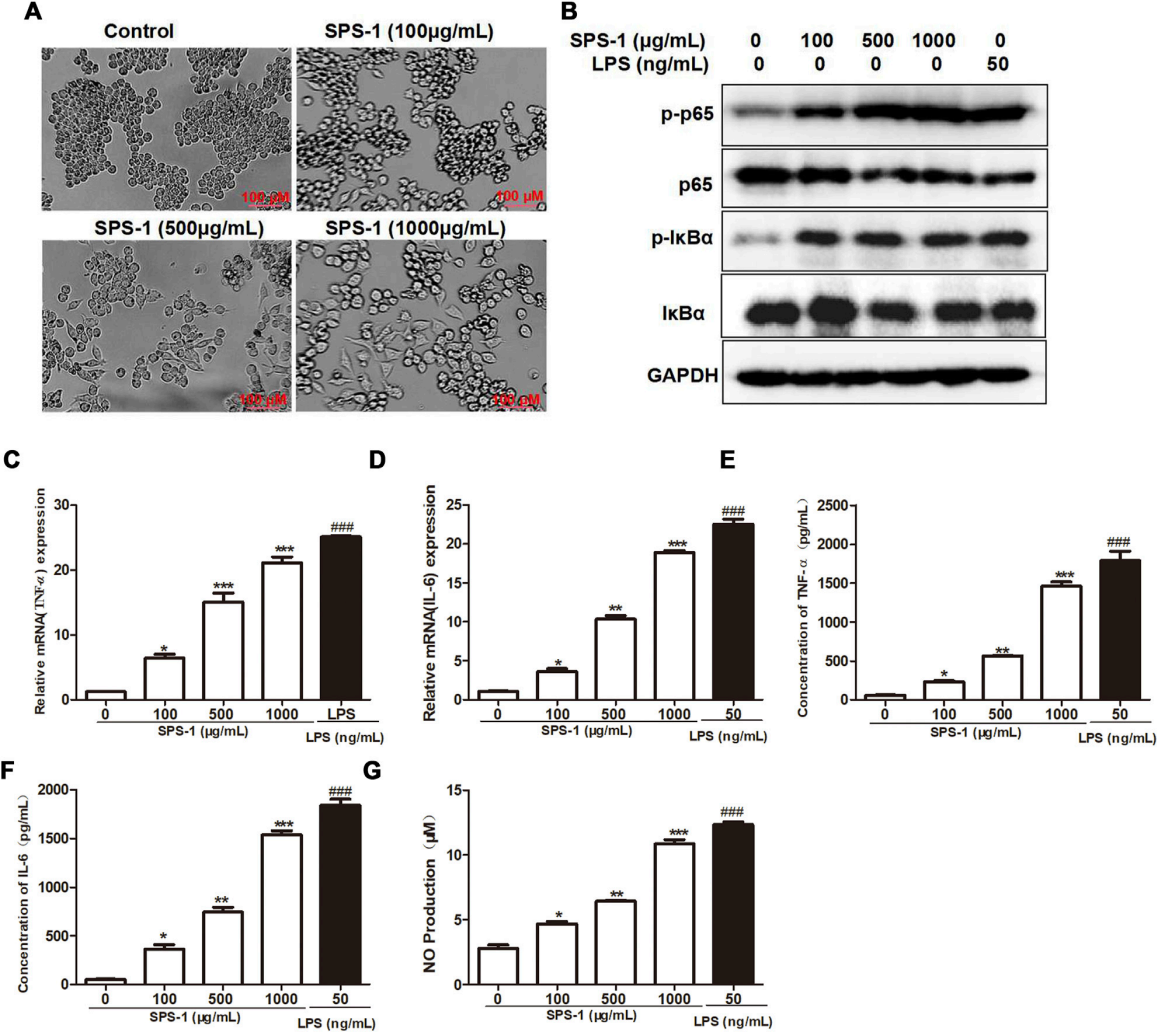
SPS-1 activated macrophages to release TNF-α, IL-6, and NO through the NF-κB signaling pathway. **(A)** Morphology of RAW 264.7 after treatment with SPS-1. **(B)** The expression of IκBα, p-IκBα, p65, and p-p65 after being treated with SPS-1. The mRNA levels of TNF-α **(C)** and IL-6 **(D)** in RAW 264.7 after being treated with SPS-1. The levels of TNF-α **(E)**, IL-6 **(F)**, and NO **(G)** in RAW 264.7 after being treated with SPS-1. Data are shown as mean ± SEM. * indicate *p* < 0.05, ** and ^##^ indicate *p* < 0.01, *** and ^###^ indicate *p* < 0.001 compared with the control group.

The change in cell morphology is an important marker for macrophage activation (Sica and Mantovani, 2021). Bright field imaging results showed that the RAW 264.7 cells morphology was significantly changed after SPS-1 treatment, together with long protrusions and pseudopodia (Figure 5A). The activation of protein kinases such as IκBα and p65 has been proven to activate macrophages (Yang et al., 2019). To examine whether NF-κB signaling was involved in SPS-1–regulated cell activation, RAW 264.7 cells were treated with increasing concentrations of SPS-1, and then the phosphorylation levels of IκBα and p65 were analyzed. As shown in Figure 5B, SPS-1 could increase IκBα phosphorylation, as well as the phosphorylation of the p65 NF-κB subunit in RAW 264.7 in a concentration-dependent manner. Subsequently, we also tested the cytokines released by macrophages after the activation of SPS-1. As shown in Figures 5C,D, the mRNA levels of TNF-α and IL-6 were significantly increased after SPS-1 treatment. Meanwhile, ELISA results showed that the secretion of TNF-α and IL-6 was also significantly increased when RAW 264.7 was treated with SPS-1 (Figures 5E,F). In addition, the production NO by macrophages was also increased significantly in a concentration-dependent manner after SPS-1 treatment (Figure 5G). These results showed that SPS-1 activated macrophages to release TNF-α, IL-6, and NO through the NF-κB signaling pathway.

### Safflower Polysaccharide-1–Activated Mφ Inhibited Colorectal Cancer Cells Proliferation and Induced Apoptosis

**FIGURE 6:**
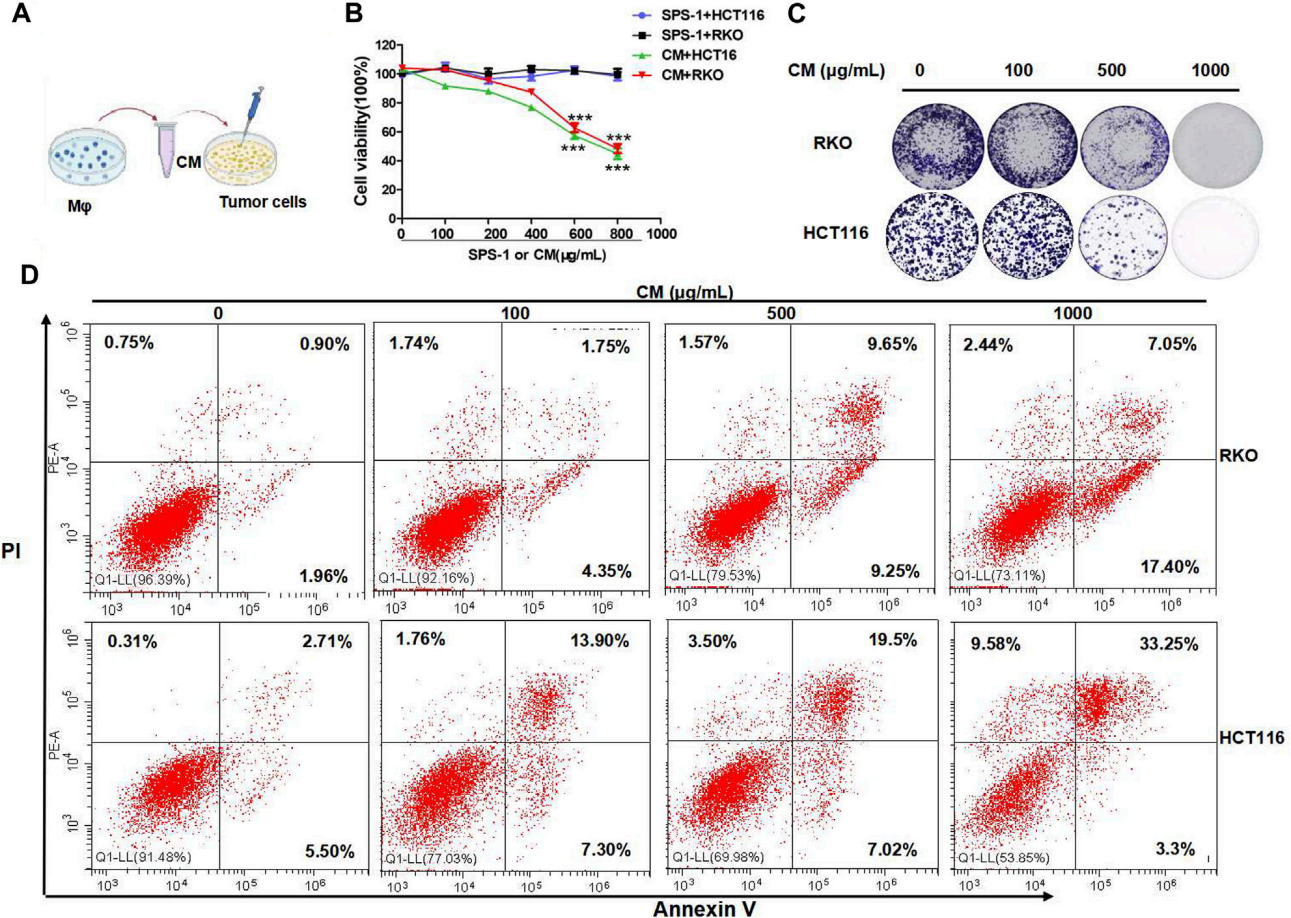
SPS-1–activated Mφ inhibited colon cancer cells proliferation and induced apoptosis. **(A)** The condition medium (CM) of Mφ was pretreated with SPS-1. **(B)** CM of SPS-1–activated Mφ inhibited the growth of RKO and HCT116 cells in different concentrations. **(C)** The effect of SPS-1 or the condition medium (CM) on the proliferation of RKO and HCT116 cells. **(D)** The apoptosis induced by CM in RKO and HCT116 cells was analyzed by flow cytometry. *** and ###indicate *p* < 0.001 compared with the control group.

**FIGURE 7:**
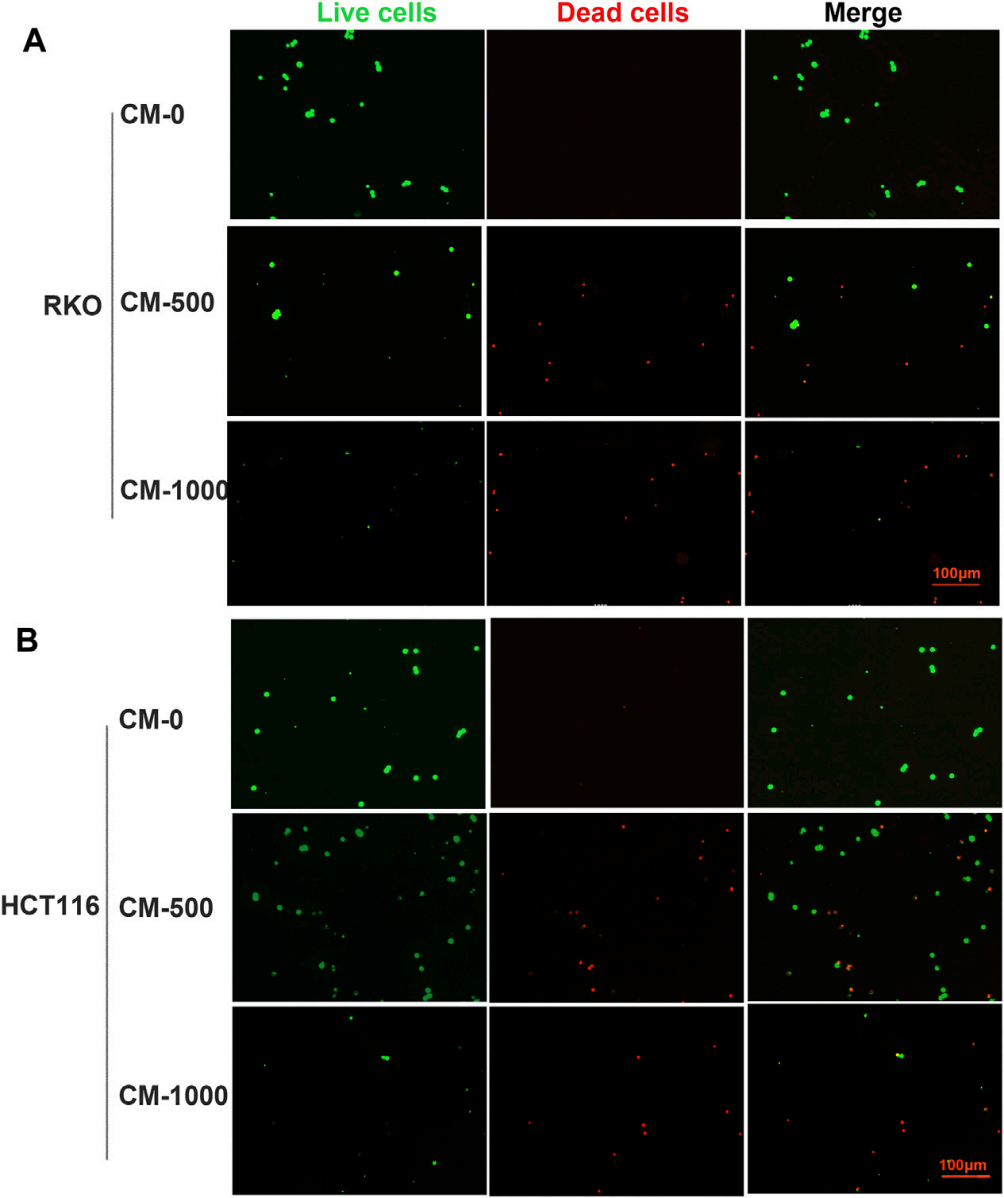
Live/dead staining evaluated the effect of CM on CRC cells apoptosis. **(A)** RKO and **(B)** HCT116 were inoculated into six-well plates (5 × 10^5^ cells/ml) containing different concentrations of CM for 72 h. Then, cells were stained with calcein-AM and PI after exposure to CM (100–1,000 μg/ml). Imaging was performed using a fluorescence tracing microscope.

To further study the antitumor activity of SPS-1–activated Mφ, the condition medium (CM) of Mφ pretreated with SPS-1 was established (Figure 6A). As shown in Figure 6B, the CM of Mφ pretreated with SPS-1 exhibited a significant inhibitory effect on the proliferation of RKO and HCT116 cells. Meanwhile, clonogenic survival of RKO and HCT116 cells after being treated with CM was measured by a colony-forming unit assay. The results showed that the colony-forming efficiency of RKO and HCT116 cells treated with CM of Mφ pretreated with SPS-1 was significantly inhibited (Figure 6C). Furthermore, the flow cytometry analysis to evaluate the role of CM of Mφ pretreated with SPS-1 in RKO and HCT116 cell apoptosis was performed. As shown in Figure 6D, the apoptotic rates of RKO and HCT116 cells were significantly increased in a concentration-dependent manner of SPS-1. Moreover, the effect of CM of Mφ pretreated with SPS-1 on CRC cells apoptosis was also determined with live/dead staining. The results showed that the numbers of live cells stained with calcein-AM were markedly decreased and dead cells with red fluorescence were significantly increased in RKO and HCT116 cells after treatment with CM (Figures 7A,B).

### Safflower Polysaccharide-1–Activated Mφ Induced Colorectal Cancer Cells Apoptosis Through the NF-κB Signaling Pathway

**FIGURE 8:**
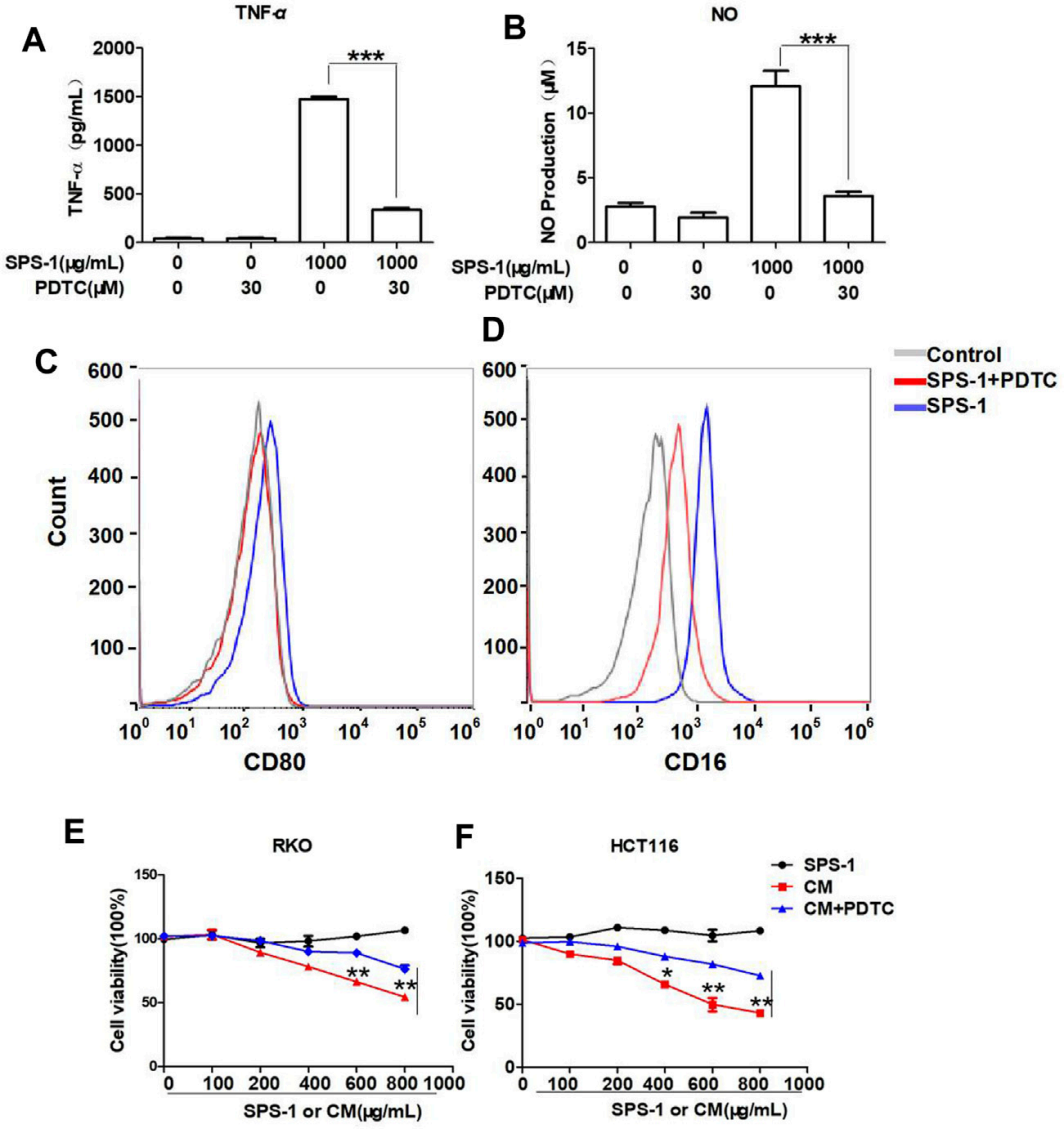
SPS-1 activated Mφ through the NF-κB signaling pathway inhibited CRC cells proliferation. RAW 264.7 cells were preincubated with or without PDTC (30 µM) for 1 h and then treated with 1,000 μg/ml of SPS-1. RNA expression of TNF-α **(A)** was tested by qRT-PCR, and the production of NO by RAW 264.7 cells was detected by the NO kit. The expression of CD80 **(C)** and CD16 **(D)** was analyzed by flow cytometry. Next, the effect of SPS-1, CM, and CM+PDTC on the proliferation of RKO **(E)** and HCT116 **(F)** cells was measured by the Cell Counting Kit-8. Data are shown as mean ± SEM. **indicate *p <* 0.01, ***indicate *p <* 0.001 compared with the SPS-1+PDTC group.

**FIGURE 9:**
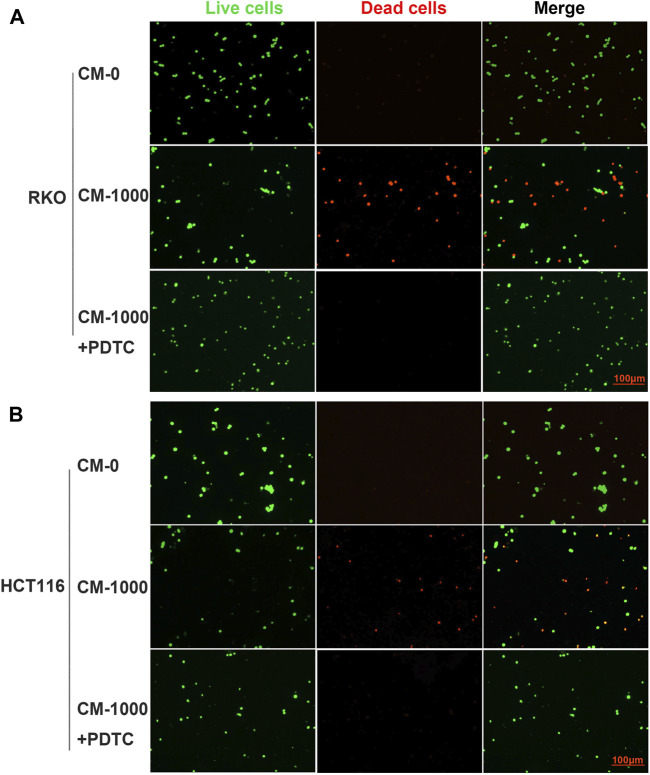
Live/dead staining evaluated the effect of CM or CM+PDTC on CRC cells apoptosis. PDTC was used to block the NF-κB pathway in macrophages and collected the condition medium (CM) of Mφ pretreated with SPS-1 or SPS-1+PDTC. **(A)** RKO and **(B)** HCT116 were inoculated into six-well plates (5 × 10^5^ cells/ml) containing different concentrations of CM. Then cells were stained with calcein-AM and PI after exposure to CM (100–1,000 μg/ml). Imaging was performed using a fluorescence tracing microscope.

To further evaluate whether SPS-1 activated macrophages through the NF-κB signaling pathway, the NF-κB inhibitor (PDTC) was used to block the NF-κB pathway in macrophages. As shown in Figures 8A,B, PDTC treatment reversed the effects of SPS-1 on the release of TNF-α and NO, and reduced the expression of CD16 and CD80 (Figures 8C,D). Furthermore, CCK-8 experiments demonstrated that PDTC could significantly reduce the inhibitory effect of CM on RKO and HCT116 cells in a concentration-dependent manner (Figures 8E,F). In addition, live/dead staining results showed that the antitumor effect of CM could be significantly reversed after pretreatment of macrophages with PDTC (Figures 9A,B). These results suggested that SPS-1–activated Mφ inhibited CRC cells proliferation and induced apoptosis mainly through the NF-κB signaling pathway.

## Discussion

In this study, we found that SPS-1, an active fraction extracted from SPS, could significantly inhibit AOM/DSS-induced CRC *in vivo*. The repeated AOM challenges resulted in increasing levels of IL-6 and TNF-α in serum and intestinal tissue, which contributes to AOM/DSS-induced CRC (Wang et al., 2019). However, the expression of these pro-inflammatory factors was not significantly reduced by SPS-1 treatment, indicating that SPS-1 did not prevent the occurrence of CRC through relieving inflammation. The tumor microenvironment is essential for tumorigenesis and development. When detecting the number of immune cells in CRC tissue induced by AOM/DSS, it was found that only the proportion of macrophages increased significantly. Therefore, we speculated that SPS-1 activation of macrophages is the main way against AOM/DSS-induced CRC.

Macrophages play important roles in the tumor immune microenvironment due to their heterogeneity and plasticity and can be polarized when affected by external microenvironmental factors (Pan et al., 2020). The blood vessel nutrition and oxygen supply in tumor tissue are insufficient during tumor growth, which leads to increased secretion of lactic acid and TGF-β and induces macrophages to differentiate into M2-type (Zhou et al., 2017). Therefore, macrophages in tumor tissues are generally M2-type and contribute to tumor proliferation, invasion, and metastasis (Katoh, 2016). However, in CRC model mice, modulating the differentiation of macrophages to M1 type could effectively inhibit tumor growth and metastasis (Chen et al., 2019). Thus, the regulation of the macrophage differentiation phenotype is one of the important research directions of CRC immunotherapy. In this research, we found that the morphology of the macrophage could be changed and induced to be of M1-type after 24 h of treatment with SPS-1, and the expression of M1-related genes was also significantly upregulated. Consistent with the *in vivo* results, *in vitro* results also demonstrated that SPS-1 could induce the polarization of macrophages to M1-type. In general, cytokines and chemokines are primary regulators of immune defense and some cytokines produced by macrophages are mediators of tumor cytotoxicity (Najafi et al., 2019). TNF-α, which is produced mainly by activated macrophages/monocytes, has the broadest activity (Cruceriu et al., 2020). Besides, NO is a kind of free radical gas, as one of the smallest and simplest biologically active molecules in nature, which can effectively inhibit the growth of tumor cells and induce apoptosis (Balkwill., 2019). The current research results showed that SPS-1 could activate macrophages with the increase of NO and TNF-α but it cannot directly induce tumor cell apoptosis. Encouragingly, SPS-1–mediated CM significantly inhibited the proliferation of cancer cells and induced apoptosis in a dose-dependent manner. These results indicated that SPS-1–activated Mφ possessed markedly antitumor activity on CRC cells.

Activation of the NF-κB signaling pathway is closely related to the polarization of macrophages (Sica and Mantovani, 2012). As previous studies, some Chinese medicines and their extracts regulate the phenotype polarization of macrophages *via* this pathway (Wang.et al., 2015). In this study, the SPS-1 could activate macrophages through the NF-κB signaling pathway. Subsequently, when macrophages were treated with the NF-κB inhibitor, PDTC, M1 polarization induced by SPS-1 was significantly inhibited. In addition, the effect of CM on tumor proliferation and apoptosis was also reversed. These results suggested that SPS-1 induced the M1-type polarization of macrophages by activating the NF-κB signaling pathway, thus exhibiting antitumor activity.

In summary, SPS-1 exhibited tumor suppressive effect by activating macrophages and inducing them to M1-type polarization through the NF-κB signaling pathway. This finding provided a new insight for the development of clinical strategies for CRC immunotherapy, which could be used alone or in combination with chemotherapy drugs to prevent the development of CRC and improve the survival of patients.

## Data Availability

The datasets presented in this study can be found in online repositories. The names of the repository/repositories and accession number(s) can be found in the article/Supplementary Material.
