# Supplementary figures and images for: Safflower Polysaccharide Inhibits AOM/DSS-Induced Mice Colorectal Cancer Through the Regulation of Macrophage Polarization
Source: Front Pharmacol. 2021 Oct 22;12:761641. doi: 10.3389/fphar.2021.761641 (PMC8569377; doi:10.3389/fphar.2021.761641)

Figure 5B


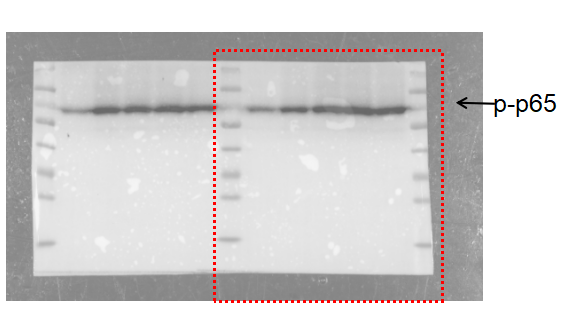


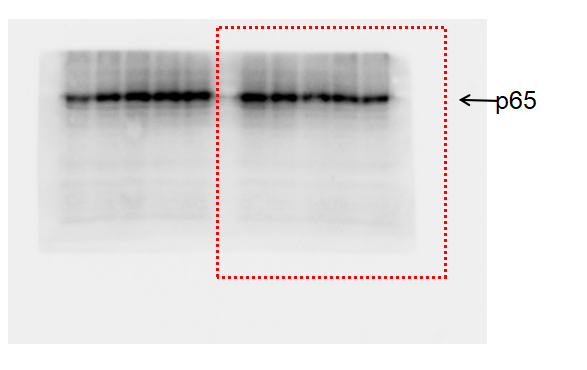


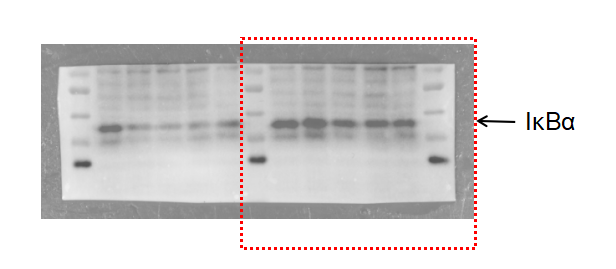


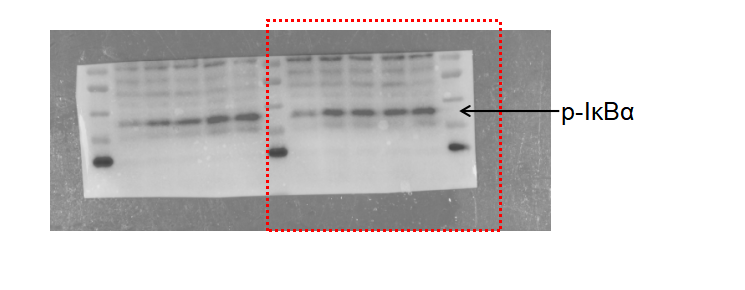


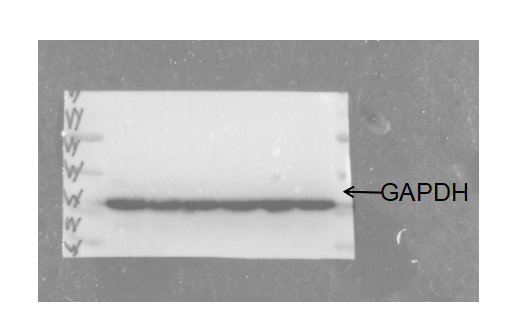

Supplement: Supplementary file 1 [file DataSheet1.DOC]
