# Supplementary material for: Safflower Polysaccharide Inhibits AOM/DSS-Induced Mice Colorectal Cancer Through the Regulation of Macrophage Polarization
Source: Front Pharmacol. 2021 Oct 22;12:761641. doi: 10.3389/fphar.2021.761641 (PMC8569377; doi:10.3389/fphar.2021.761641)
Supplement: Supplementary file 2 [file DataSheet2.doc]

**Safflower polysaccharide inhibits AOM/DSS induced mice colorectal cancer through the regulation of macrophage polarization**

**Qun Wang^a†^, Yun Huang^a†^, Min Jia^b^, Dong Lu^a^, Hong-wei Zhang^a^，Dan Huang^c*^, San-hong Liu^a*^, Chao Lv** **^a*^**

^a^ Institute of Interdisciplinary Integrative Medicine Research, Shanghai University of Traditional Chinese Medicine, Shanghai 201203, China

^b^ College of Pharmaceutical Sciences, Zhejiang Chinese Medical University, Hangzhou 310053, China

^c^ Department of Pathology, Fudan University Shanghai Cancer Center, Shanghai 200032, China

**1. Material and methods**

The crude polysaccharides were collected and loaded into a DEAE Sepharose Fast Flow column (1.6cm × 20cm, General Electric Healthcare Life Sciences, Fairfield, Connecticut, USA) to obtain the fraction of major peak SPS-1. The average molecular weight (Mw) of SPS-1 were determined by high-performance size exclusion chromatography (HPSEC). The monosaccharide composition was analyzed by a Shimadzu Lc-10Avp system, Shimadzu RID-10 A detector (Shimadzu, Kyoto, Japan) and Aminex HPX-87 P column (Bio-Rad, Hercules, California, USA). For the infrared spectrum (IR) analysis, the dry SPS-1 powder and potassium bromide were mixed in the mass ratio of 2:98 and tableted to a translucent sheet, which was then scanned 32 times at 4000–400 cm^−1^ using a Fourier Transform infrared spectrophotometer (Thermo Scientific, Waltham, MA, USA).

**2 Results**

**2.1 Chemical properties of SPS-1**

Based on the phenol-sulfuric acid method, the elution curve (Supplementary Figure 1) indicated that SPS was separated by DEAE-cellulose 52 to obtain four components with good resolution: 0.1 mol/L NaOH elution fraction SPS-2, 0.2 mol/L NaCl elution fraction SPS-3, 0.3 mol/L NaOH elution fraction SPS-1, and 0.4 mol/L NaOH elution fraction SPS-4.The major components of AP were arabinose (36.06%), mannose (6.1%), glucose (44.37%), and galactose (13.48%) (Supplementary Figure 2). SPS-1 were obtained, with a molecular weight of 4.1 KDa (Supplementary Figure 3) as evaluated by Ohpak SB-804 gelatin column. The IR spectrum of SPS-1 (Supplementary Figure 4) exhibited absorption bands at 3394, 2935, 1627 and 1415 cm^−1^, attributing to O-H stretching vibration, C-H stretching vibration, C-O stretching vibration and C-H bending vibration, respectively. The four bands above were characteristic absorption bands of polysaccharide. The bands at 1101cm^−1^ indicated the pyran configurations of polysaccharides .


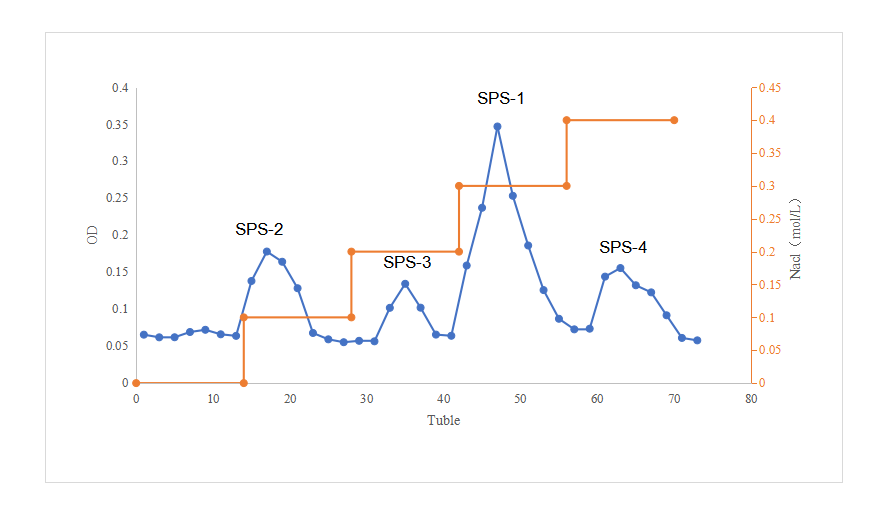


**Supplementary Figure 1** DEAE elution profile of SPS-1


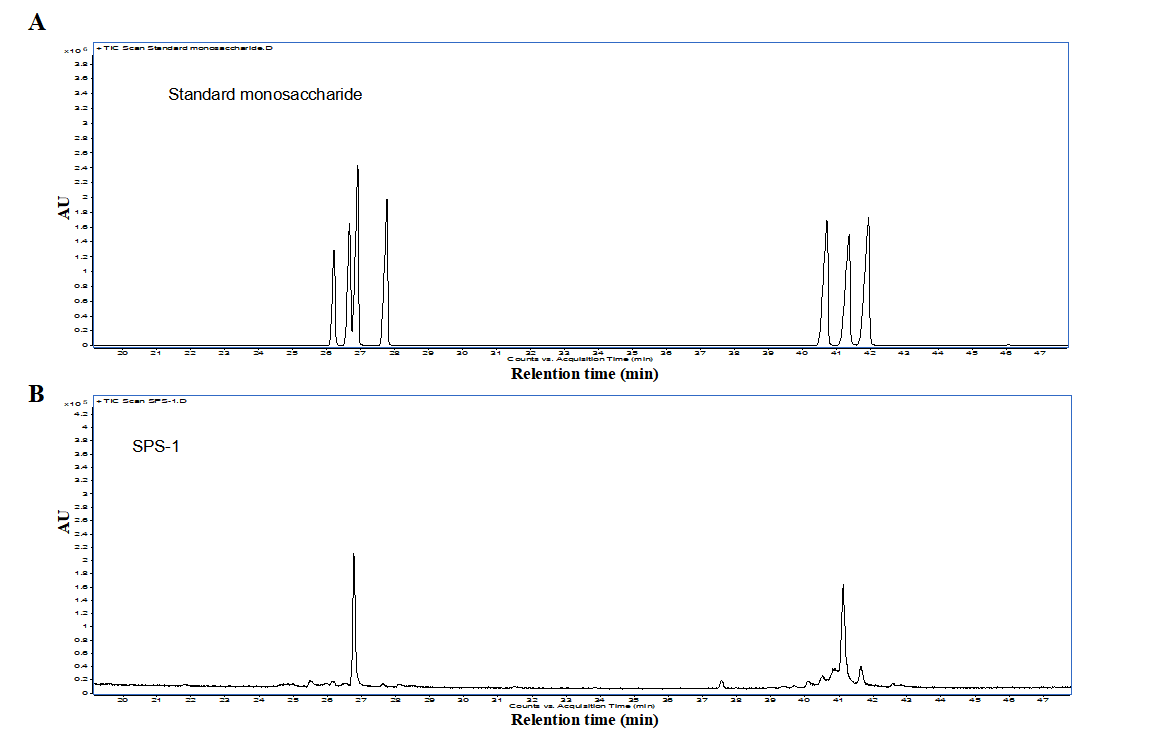


**Supplementary Figure 2** Monosaccharide composition analysis by HPLC, (A) chromatogram of standard monosaccharide, rhamnose, fucose, arabinose, xylose, mannose, glucose, galactose, (B) chromatogram of monosaccharide composition in SPS-1.


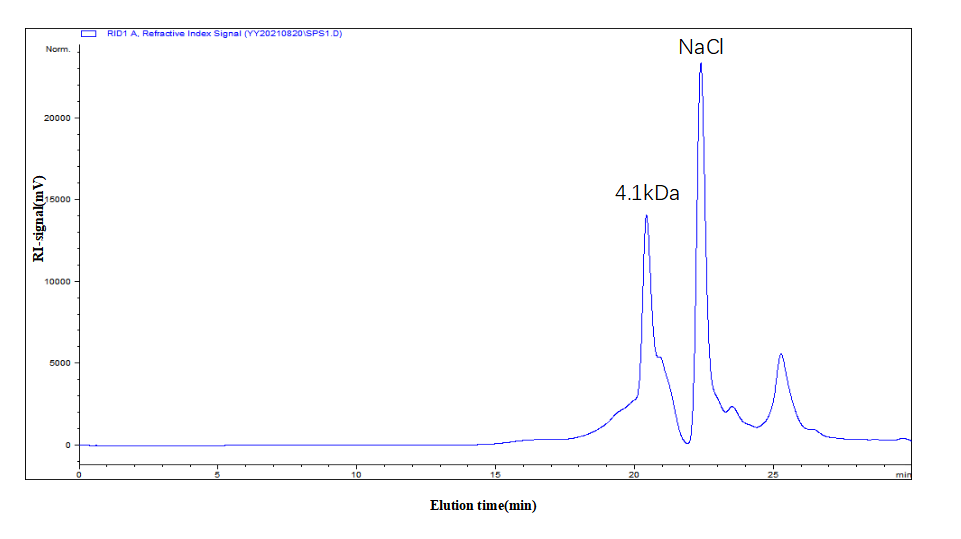


**Supplementary Figure 3** HPSEC elution profile of SPS-1


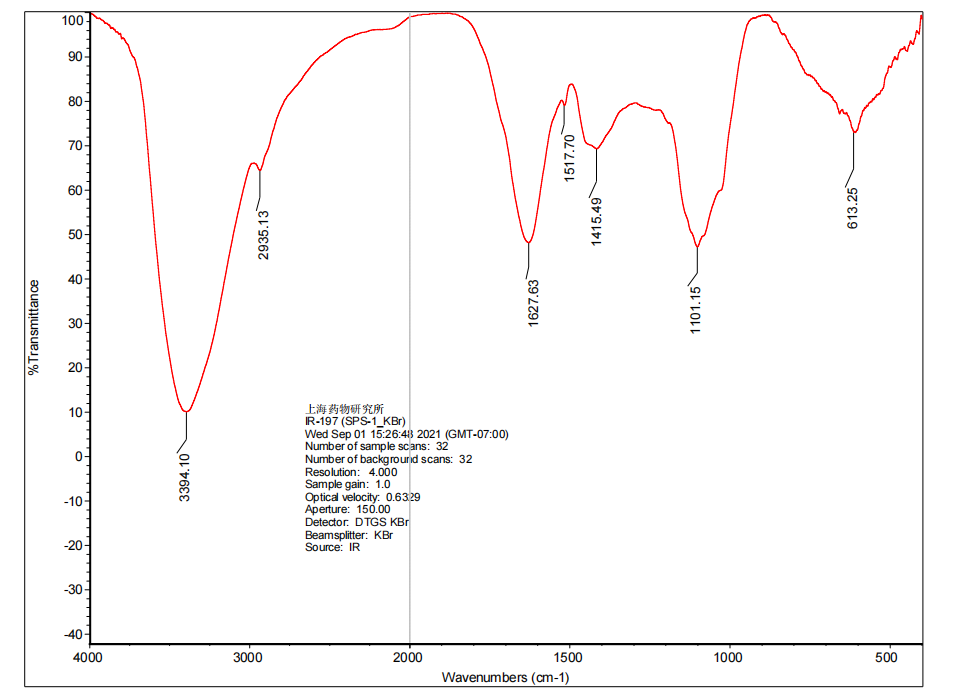


**Supplementary Figure 4** Characterization of SPS-1 by FT-IR spectrum.
